# PReS-FINAL-2032: Changes in immunogenic profile in patients with juvenile idiopathic arthritis exposed to anti tumoral necrosis factor therapy

**DOI:** 10.1186/1546-0096-11-S2-P45

**Published:** 2013-12-05

**Authors:** CA Guillen Astete, WA Sifuentes Giraldo, ML Gamir

**Affiliations:** 1 Pediatric Rheumatology Unit, Ramon y Cajal Universitary Hospital, Madrid, Spain

## Introduction

In a previous study conducted by our group we observed an unusual prevalence of tittles of antinuclear (ANA) and anti-DNA (aDNA) antibodies in children who were previously negative for both antibodies and who received any biologic therapy due to a childhood rheumatic systemic disease (CRSD). This observation has been reported in adulthood previously, mainly in patients exposed to anti tumoral necrosis factor (aTNF) therapy however its clinical impact is not well established.

## Objectives

Present study was conducted to determine if aTNF therapy administered on patients with Juvenile idiopathic arthritis (JIA) shows the same tendency and if the ratio of change is comparable to patients not exposed to this therapy.

## Methods

Medical records of our cohort of patients from 1999 to 2009 who underwent 62 an aTNF therapy due to any ILAR category of JIA were reviewed. Analytical tests were collected in the moment of diagnosis, at the beginning of the aTNF treatment, and at the 3rd and 6th month of treatment. Only cases with complete analytical testing up to a month after the designed date were analyzed. Comparisons were made with children diagnosed with the same ILAR category of JIA of the same age (+/- 2 years old) and sex. Non exposed and exposed subjects ratio was 1:1. aDNA positive change was considered when a negative cuantitative determination changed to a positive one according to our reference lab ranges (Indirect immunofluorecense method). ANA increase was defined as a two fold increase in the titter. In order to establish comparisons only subjects susceptible to experiment an ANA titter increase of an aDNA positive change were chosen.

## Results

The followed up treatments were: 32 Etanercept (ETN), 15 Infliximab (IFM), 15 Adalimumab (ADM), Anti-DNA profile evolution: 3/25 showed a titter increase (12.0%), all of them were previously negative (2 with ETN and 1 with IFM). Into the control group no patient developed a titter increase (p non available). ANA profile evolution: 12/49 showed a titter increase (24.4%), 8 with ETN and 4 with IFM. Into the control group 5/49 patients (10.2%) experiment a titter increase (p < 0.05). No patient who underwent a change into its autoimmune profile was secondarily diagnosed with other CRSD during the period of follow up. The need of switching the biologic therapy, rate of severe complications or demands of increase of non biologic therapy was similar in children who increase its ANA titters and who did not.

## Conclusion

Our findings supports the hypothesis that aTNF therapy plays a role into the change of the autoimmunity profile in children with JIA. The most important fact around this findings is related to the clinical repercussion. Although we could not demonstrate any significant clinical impact it is possible that longer periods of observation in larger series can, so the close observation of the autoimmunity profile should be, in the meantime, part of the follow up of children with JIA exposed to aTNF therapy.

## Disclosure of interest

None declared.

